# A Communication Concerning the Eruptions Resembling the Small-Pox, Which Sometimes Appear in the Inoculated Vaccine Disease

**Published:** 1800-02

**Authors:** George Pearson

**Affiliations:** Physician to St. George's Hospital, &c.


VOL. III.] FEBRUARY, 1800. [NO. XII.

### *A Communication concerning the Eruptions resembling the Small-Pox, which sometimes appear in the inoculated Vaccine Disease; by* GEORGE PEARSON, M.D. F.R.S. *Physician to St. George's Hospital, &c.*

FOR reasons, the explanation of which would lead into too long a detail, and which, indeed, cannot be given with perfect propriety in this sheet, I feel myself compelled to publish a few observations concerning the eruptions which appear, in some instances, in the cow-pock by inoculation.

Although the new inoculation in the present year has been, I think, sufficiently extensive to manifest the advantages of it over that of the small-pox, so that it is not likely to be ever laid aside; yet, the unexpected appearance of eruptions has inclined many persons to be of opinion, that no beneficial consequences can be produced by this practice; or, at least, that such consequences, at best, seem to be problematical. It may be useful to observe, that some of the advocates for the cow-pock inoculation contend, that eruptions are never produced by it; accordingly they assert, that in these eruptive cases the disorder was not the cow-pock, but the small-pox; the variolous poison having either been inserted inadvertently, or the constitution having been affected by it casually. To justify what is advanced, it is incumbent on the Assertors, either to prove that such errors have been committed; or, at least, they ought to be able to oppose equally extensive experience to that of the adverse party; for no sound reasoner will consider opinions which are only founded on conjecture to be demonstrated truths. I will not, however, take upon myself the unrequired task of attempting to vindicate others from the above charges, but I shall only perform a duty in stating the result of my own experience with regard to the point in question; conceiving, that by this means, the truth may be brought to light, in conjunction with the evidence from experience of future Inquirers.

In the courfe of my practice, the latter end of February and in March following, I diftinctly recollect four cafes, in which I firft faw eruptions from the vaccine inoculation, refembling fo much thofe of the fmall-pox, that I fhould not have hefitated to confider them as belonging to this difeafe, if I had not excited them by a different poifon from the variolous. I obferved, however, at that time, fome appearances of thefe eruptions different from thofe which ufually occur in the fmall-pox. Almoft all thefe eruptions, in the ftage of deficcation, afforded fhining, fmooth, black, or reddifh-brown fcabs; very few of them having previoufly fuppurated. Finding, in feveral inftances, that the matter from the inoculated puftule of thefe patients produced a fimilar eruptive diforder, and alfo the fame being the event in the practice of two or three of my correfpondents, whom I had furnifhed with matter from the above eruptive cafes, I, from that time, avoided ufing matter from the cafes in which fuch eruptions appeared. After this precaution, no eruptive cafes refembling the fmall-pox, but certainly eruptions, in number from a fingle one to about a dozen, which were large, red, hard pimples, with little or no lymph, and never with any pus, occurred in probably one cafe out of twenty or thirty. Thefe fpots, fo unlike the fmall-pox, gave no trouble, and were of fuch a fhort duration, that when I fpeak of eruptions I do not include them in the account; I include thofe only in which the eruptions refembled the fmall-pox: nor do I reckon among the eruptive cafes, thofe in which, in a few inftances, a rafh broke out about the 14th day after inoculation, and which was as troublefome as the *Urticaria*. My experience then, with refpect to the cafes of eruptions, being diminifhed in number, by avoiding inoculation with matter of fimilar eruptive cafes, coincides with Dr. Woodville's, and confirms what he has already fo ufefully communicated to the public.

It was obvious to fufpect, on the firft occurrence of the eruptive cafes, that variolous matter in an obferved way, but from the fources which could not even be conjectured, had been introduced into the conftitution inftead of the vaccine poifon. This conjecture, in fpite of the cleareft evidence of fenfe refpecting the nature of the matter ufed, received fome countenance from the non-appearance of eruptions as above ftated; but from the occurrence of fuch cafes in the practice of other Inoculators in the laft autumn and this winter, I now think it very unreafonable to doubt any longer, that, either on account of peculiar ftates of the human animal œconomy, or on account of fome co-operating agents, the genuine vaccine poifon does occafionally produce a certain variety of the cow-pock, characterized by the appearance of puftules like thofe of of the variola. I have good evidence also to shew, that even in the hands of those very Inoculators, who, a little time ago, would not allow that the vaccine poison could produce eruptions, such cases have lately occurred.

In the month of October last I inoculated a child, two years of age, with the vaccine poison. The original matter which had produced this matter, I took from the cow in March last; since which time the vaccine disease had been excited by it, in my hands, in a great number of patients.

The vaccine disease took place with the usual appearances in the inoculated part, and affected the whole constitution in the ordinary manner; but a few eruptions broke out on the second or third day, after a slight fever; they were, however, only the red large pimples aforementioned, and, of course, not at all like the small-pox. Mr. Keate carried matter from this child to Brighthelmstone, where Mr. Barrett inoculated two children, who took the disease, and from one of these Mr. Keate inoculated three children. They all had the usual fever about the eighth day, and all had a number of eruptions except one, who had only five or six, and those dried on the fifth day. This last case was probably that which Mr. Keate informed me had in the inoculated part the genuine vaccine pustule; but in all the others, Mr. Barrett observed, that in the inoculated part the pustule was ragged at the edges, and flat, most resembling the variolous pustule. Matter from these patients was sent to Petworth, where Mr. Andre informs me, he inoculated with it fourteen children. They all took the disease, and had eruptions like the variolous. Three children at the breast had from three to twelve pustules, the remaining eleven children had from fifty to several hundred eruptions. The state of the arms, and the characters of the pustules of the inoculated part are not mentioned. None of the above patients died; nor is any mention made by Mr. Keate, Mr. Barrett, and Mr. Andre, even of any apprehension of danger. I add, that about a month ago, Dr. Thornton sent the case with eruptions, produced by matter which I originally took from the cow.

According then to experience, we draw these conclusions: 1st, That in certain constitutions, or under the circumstances of certain co-operating agents, the vaccine poison produces a disease resembling the small-pox; and, of course, the pustule in the inoculated part is very different from that of the vaccine pock ordinarily occurring, and the eruptions resemble very much, if not exactly, some varieties of the small-pox.

2d. That in some instances these eruptions have occurred, although the inoculated part exhibited the genuine vaccine pustule.

3d. That the matter of such eruptive cases, whether taken from the inoculated part, or from other parts, produces universally, or at least generally, similar eruptive cases; and has not, I believe, been seen to go back, by passing through different constitutions to the state in which it produces, what is called the genuine vaccine disease.

4th. That eruptions of a different appearance from variolous ones, sometimes occur in the true cow-pock.

Now, whether the vaccine poison, when it produces these cases resembling the small-pox, really becomes by composition or decomposition, variolous matter, is undetermined. If this should be found to be the case by future experiments, still we must consider the two poisons as of distinctly different species, on account of the different characters of the pustule in the small-pox and cow-pock: although, as just said, by the combination of some other substance with the cow-pock poison, or by the separation of some one of the constituent ingredients of the poison, the variolous may be produced. To illustrate this theory, let it be considered, that magnesia and sulphate of magnesia are different species of substances, although they agree in some of their principal effects on the human constitution, and in some other properties; but, by the union of magnesia with sulphuric acid, it becomes sulphate of magnesia: or, the illustration may be given conversely. As, then, we have distinct denominations for these two substances, so we ought to have them for the two poisons, and the two different diseased states they produce, namely, the cow-pock and the small-pox. Accordingly, Dr. Odier, of Geneva, whose powers as a Dialectician, and whose acuteness as a Philologer, I can attest from the period of his academical studies, has "baptized" (to use the term of Dr. De Carro) the new disease, *la vaccine*, or *vaccina*; rejecting, as absurd, the name of the English, *variolæ vaccinæ*.

But, to return to the immediate question under discussion; granting, that variolous-like eruptions are liable to be produced by the inoculation of the cow-pock; what difference ought this accident to make in our estimate of the value of the new practice, from the estimate made on the supposition, that no such eruptions would occur? I apprehend the value is hereby depreciated, but not to such a degree as to create any reasonable apprehensions of the failure of the vaccine inoculation, in superseding and finally extinguishing the small-pox; because,

1. If the precaution be taken of avoiding inoculation with matter from eruptive cases, as far as I have seen, not above one one cafe with variolous-like eruptions will be produced among two hundred inftances of inoculated cow-pock.

2. Thefe eruptive cafes, as far as I have obferved, are not more fevere than the ordinary kinds of inoculated fmall-pox.

3. I have feen no disfiguration of the fkin from this variety of cow-pock; but I think it juft to acknowledge, that from the experience I have had, no Practitioner can anfwer for fuch cafes not occurring in any inftance; and, as danger is always in proportion to the number of eruptions, which number, no perfon can pretend to limit; it is evident, that the chance of life during this difeafe is leffened, although but a very little. Provided this ftatement be made to the patient, it does not appear to me, that the fact of the liability to eruptions ought to impede the progrefs of the vaccine inoculation; but if, on the contrary, the patient be affured that fuch eruptions will not occur, there is good ground for the practice falling into difcredit, or at leaft, with reafon, for many perfons being difcontented. After this reprefentation of an unfavourable part of the hiftory of the cow-pock, it is confolatory to be able to counterpoife it with fome new facts, which, like that of the eruptions, have been difcovered in the courfe of further experience. It now appears, that a perfon who has had the fmall-pox, is not fufceptible of the cow-pock by inoculation;* nor is a perfon fufceptible of the conftitutional affection from the cow-pock poifon more than once. On the whole, then, we have gained, perhaps, as much as we have loft, fince the publication of the original account. And, unlefs fome new adverfe facts fhall be difcovered, and confiding that the public will adopt a method which is manifeftly to their intereft, the change effected in medical practice *will be fo eminently memorable, that the introduction of the vaccine inoculation muft become an epoch in the hiftory of phyfic.*

---

### *To the Editors of the Medical and Phyfical Journal.*

GENTLEMEN,

IN your Journal for the prefent month, I obferve an extract from a paper fent you by Dr. Pearfon, on the appearance of puftules

---

* The cafes of milkers, with chopt hands, being repeatedly affected with fores from the cow-pock poifon, applied in milking, whether they had undergone the fmall-pox or not, probably occafioned the error of the conclufion here alluded to.

